# Effects of an Electric Current on the Superplastic Deformation Behavior of 3Y-TZP in an Oxygen-Lean Atmosphere

**DOI:** 10.3390/ma16206785

**Published:** 2023-10-20

**Authors:** Kang Wang, Yufei Zu, Guoqing Chen, Xuesong Fu, Wenlong Zhou

**Affiliations:** 1 Key Laboratory of Solidification Control and Digital Preparation Technology (Liaoning Province), School of Materials Science and Technology, Dalian University of Technology, Dalian 116085, China; wangkang245@mail.dlut.edu.cn (K.W.); xsfu@dlut.edu.cn (X.F.); wlzhou@dlut.edu.cn (W.Z.); 2 Key Laboratory of Advanced Technology for Aerospace Vehicles (Liaoning Province), School of Aeronautics and Astronautics, Dalian University of Technology, Dalian 116085, China; yfzu@dlut.edu.cn

**Keywords:** 3Y-TZP ceramic, electric current, plastic deformation, the activation energy

## Abstract

The aim of this paper is to investigate the mechanism of an electric current-assisted superplastic deformation on 3Y-TZP in an oxygen-lean atmosphere. The experiments were performed with different electric currents in the range of 0~5 A. The results show that the flow stress of 3Y-TZP during the deformation was significantly decreased by the combination of Joule heating and the applied current effect. The microstructures of the deformed specimens were all equiaxed grains without an obvious preferential grain growth. The stress exponent *n* = 2.05~2.61 suggested that the dominant deformation of 3Y-YZP with/without the electric current was grain boundary sliding at 1400 °C. The activation energy of the deformation which decreased from 465 kJ mol^−1^ to 315 kJ mol^−1^ by the electric current indicated that the lattice diffusion of Zr cation during the deformation was enhanced. And the deformation rate of 3Y-TZP with the electric current may be controlled by the grain boundary diffusion of Zr cation.

## 1. Introduction

Yttria-stabilized tetragonal zirconia (3Y-TZP) is generally considered a suitable candidate material in the aircraft engine and aerospace fields as a result of their excellent properties [1,2,3]. What it lacks, however, is fracture toughness and machinability to a desired shape. In recent years, some structure ceramics with grain sizes of less than 1 μm exhibited superplasticity, which the tensile elongation exceeded 100% through grain boundary sliding (GBS) [3,4,5,6,7]. Hence, superplastic forming is expected to be an effective way for fabricating ceramic components having complex shapes. 

Recently, the electric field, which could affect the diffusion transport in ceramics in a significant way, was used for improving the superplastic deformation of the structural ceramics as a result of the low strain rate during deformation [8,9,10,11,12,13,14,15]. At different electric fields, work by Conrad and co-workers [8,9,10,12] on yttria-stabilized tetragonal zirconia in air showed a significant enhancement in ductility. They also discovered that the flow stress with the electric field can be decreased by retarding the grain growth. However, all these experiments were operated at temperatures above 1400 °C and strain rates below 10^−4^ s^−1^, which would make it difficult to achieve the industrial production of structural ceramics.

More recently, Raj and coworkers [15,16,17] reported that the sintering of yttria-stabilized zirconia can be densified rapidly in a few seconds with high DC electric fields (100 Vcm^−1^) at low furnace temperatures (1000 °C). They believed that the applied electric field on yttria-stabilized zirconia may accelerate the diffusional transport during the sintering. Yoshida et al. [13,18,19] studied the superplastic deformation behavior of ZrO_2_ under flash sintering conditions and discovered that the superplasticity in ZrO_2_ can only be improved by the electric current within a certain range. They believed that the decreased flow stress by the electric current was attributed to a combination of the current-induced increase in diffusion and Joule heating. Meanwhile, Luo and coworkers [20] discovered that a reduced atmosphere can effectively promote the flash sintering of ZnO to achieve nearly full densities at a furnace temperature below 120 °C. Hulbert et al. [21] also discovered that a high strain rate formable in ZrO_2_-Al_2_O_3_-spinel composite ceramics can be achieved by using spark plasma sintering (SPS) equipment at a furnace temperature of 1100 °C. They believed that this high-speed superplastic forming was made possible by taking advantage of the enhanced diffusional transport in the SPS chamber environment. Furthermore, Dong and Chen [22,23] reported that grain growth in zirconia can be accelerated in a N_2_ + 5%H_2_ atmosphere by an electric current. They pointed out that this remarkable grain growth was related to the enhancement of cation diffusion in an oxygen-lean atmosphere. Therefore, an oxygen-lean atmosphere will inevitably affect the deformation behavior and mechanism of the electric field/current-assisted superplastic deformation on ZrO_2_.

It is well-known that the deformation behavior and the corresponding mechanism for structure ceramics can be identified by the following constitutive equation:

(1) $$\dot{\varepsilon }=A\sigma^{n}d^{-p}exp\left(-\frac{Q}{RT}\right)$$

where *A* is a constant, $\dot{\varepsilon }$ is the steady state strain rate, σ is the flow stress, d is the average grain size, *n* is termed the stress exponent, the parameter *p* is the grain size exponents, and the *Q*, *R*, and *T* are the activation energy, gas constant, and absolute temperature, respectively.

Wakai, F. et al. [1,24,25] investigated the superplastic behavior of ZrO_2_ polycrystals and suggested that the mechanism for superplastic deformation was grain boundary sliding, which was characterized by a stress exponent of 2. In addition, Jimenen-Melendo et al. [26,27] studied superplastic deformation in the fine-grained 3Y-TZP and proposed that the mechanism for deformation was grain boundary sliding accompanied by a dislocation motion. This mechanism was approved by Morita et al. [28], and they believed that the deformation rate is controlled by the rate of dislocation recovery. The superplastic deformation of 3Y-TZP with an initial grain size of around 135~145 nm was investigated by Bernard-Granger et al. [29]. They found that the stress exponent of the deformation was about 3, and they suggested that the dominant deformation mechanism was still grain boundary sliding as a result of the microstructure with equiaxed grains. Therefore, grain boundary sliding was generally considered as the dominant deformation mechanism for the superplastic deformation of 3Y-TZP at present. The reports for the electric field-assisted superplastic deformation of 3Y-TZP worked on by Conrad and co-workers [8,9,10] indicated that the dominant mode of deformation for 3Y-TZP was not the electric field but was still grain boundary sliding. However, there are few research works concerning the behavior and mechanism of the electric field-assisted plastic deformation on zirconia ceramic in an oxygen-lean atmosphere.

Therefore, the present work aims to investigate the mechanisms of the electric current-assisted plastic deformation on 3 mol% Y_2_O_3_ stabilized tetragonal ZrO_2_ ceramics (3Y-TZP) in an oxygen-lean atmosphere. For this purpose, the flow behavior of 3Y-TZP was examined at different initial strain rates and temperatures.

## 2. Experimental Procedures

**Figure 1:**
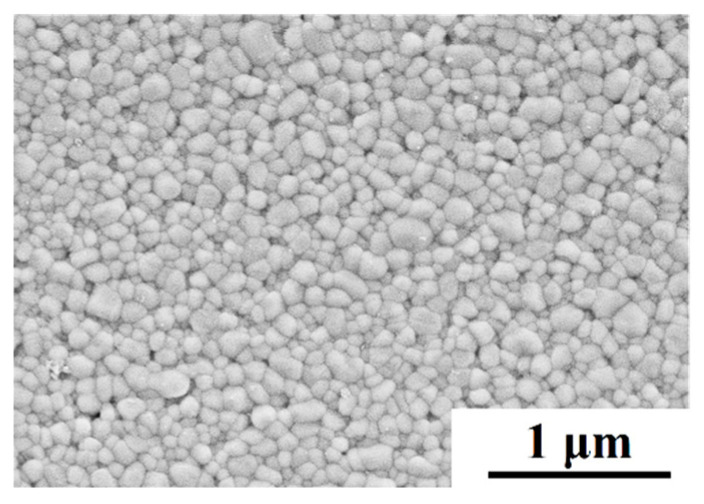
The microstructure of sintered zirconia at 1200 °C.

The starting materials used in this paper were powders of TZ-3Y, with a particle size of 27 nm (Tosoh Co., Tokyo, Japan). The specimens for the deformation were sintered by using these powders at 1200 °C, a 30 MPa applied pressure, and at a 1 h holding time in a Hot Press Sintering Furnace. The relative density of the as-sintered specimen was 99.3%, which was measured by the Archimedes method. The geometry of the as-sintered 3Y-TZP for the compression deformation was cylindrical, with 10 mm in diameter and 10 mm in height. The SEM micrographs of the as-sintered 3Y-TZP specimen are shown in Figure 1. The average grain size of the as-sintered 3Y-TZP specimen is 125 nm.

The deformation experiment was operated by self-designed equipment. In order to ensure the electric current only passed though the specimen, a special graphite with an alumina tube wan was designed. The upper/lower graphite indenter (electrode) was connected with a voltage-stabilized DC power supply device by using electric wires. The schematic diagram of the apparatus used for the experiment is shown in Figure S1 (Supplementary Information). The deformation temperatures were set to 1200 °C, 1300 °C, and 1400 °C, and the furnace was heated at a rate of 10 °C·min^−1^ until it reached the set temperature. In order to guarantee a uniform temperature, the specimens were kept at the deformation temperature for 10 min before the deformation. The experiment was performed at different electric currents: 1 A, 3 A, and 5 A. The corresponding initial current densities through the specimen were 12.7 mA/mm^2^, 38.2 mA/mm^2^, and 63.7 mA/mm^2^. The temperature of the furnace and specimen were both measured and recorded by the deformation device. For each applied electric current, the immediate height *H_x_* and the load *P* were recorded by adjusting the punch rates at 0.1, 0.2, and 0.4 mm·min^−1^, respectively. The initial end face area *A*_0_ and the initial height *H*_0_ of the cylindrical specimen were directly measured. The true stress σ, the true compressive strain ε, and the strain rate $\dot{\varepsilon }$ of the specimens were expressed as follows:

(2) $$\varepsilon =-ln\left(\frac{H_{x}}{H_{0}}\right)$$

(3) $$\sigma =\frac{{PH}_{x}}{A_{0}H_{0\;}}$$

(4) $$\dot{\varepsilon }=\frac{{H_{x}-H}_{x-1}}{H_{x}}$$

The specimens were polished by a diamond paste of 1.5 μm in diameter and thermally etched for 100 min at a temperature 50 °C lower than the sintering and/or deformation temperatures in an oxygen atmosphere. The microstructures of the specimens were characterized by a scanning electron microscopy (SEM) system (S-4300, Hitachi, Tokyo, Japan). The average grain sizes of the deformed specimens were calculated by using the linear intercept method.

## 3. Results and Discussion

**Figure 2:**
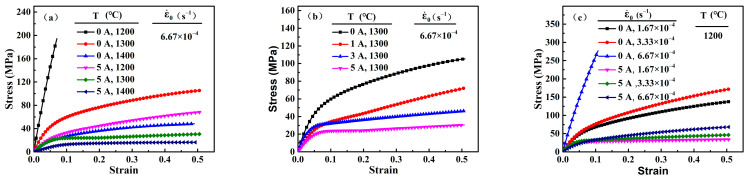
The true stress-strain data of 3Y-TZP with and without the electric currents (I) at the different furnace temperatures (T) and initial deformation rates (${\dot{\varepsilon }}_{0}$). (**a**) ${\dot{\varepsilon }}_{0}$: 6.67 × 10^−4^ s^−1^, *T*: 1200 °C, 1300 °C and 1400 °C, *I*: 0 A and 5 A; (**b**) ${\dot{\varepsilon }}_{0}$: 6.67 × 10^−4^ s^−1^, *T*: 1300 °C, *I*: 0 A, 1 A, 3 A and 5 A; (**c**) ${\dot{\varepsilon }}_{0}$: 1.67 × 10^−4^ s^−1^, 3.33 × 10^−4^ s^−1^ and 6.67 × 10^−4^ s^−1^, *T*: 1200 °C, *I*: 0 A and 5 A.

The stress-strain curves of 3Y-TZP specimens with and without the electric current are shown in Figure 2. The data of the flow stress in Figure 2a at 0 A and 1200 °C, 0 A and 1400 °C, and 5 A and 1400 °C were obtained from a previous study [30]. As shown in Figure 2a, the specimen hardly deformed with the initial strain rate of 6.67 × 10^−4^ s^−1^ at a furnace temperature of 1200 °C. And the flow stress for the specimen at 1300 °C and 1400 °C were 105.44 MPa and 48.52 MPa, respectively. By contrast, the flow stress of deformation decreased sharply as the electric current was applied. The flow stress of the specimen with the electric current of 5 A at 1200 °C, 1300 °C, and 1400 °C were 63.59 MPa, 30.72 MPa, and 18.25 MPa, respectively. The flow stress decreased from approximately 75 MPa to 30 MPa. In addition, with the electric current increased, the flow stress decreased significantly and the apparent strain hardening was eliminated (Figure 2b), which was similar to a previous study at 1200 °C [30]. The deformation of 3Y-TZP at different initial deformation rates can also be promoted by the electric current (Figure 2c). These results indicate that the electric current was instrumental in decreasing the flow stress and the furnace temperature during the superplastic deformation of 3Y-TZP in an oxygen-lean atmosphere.

**Figure 3:**
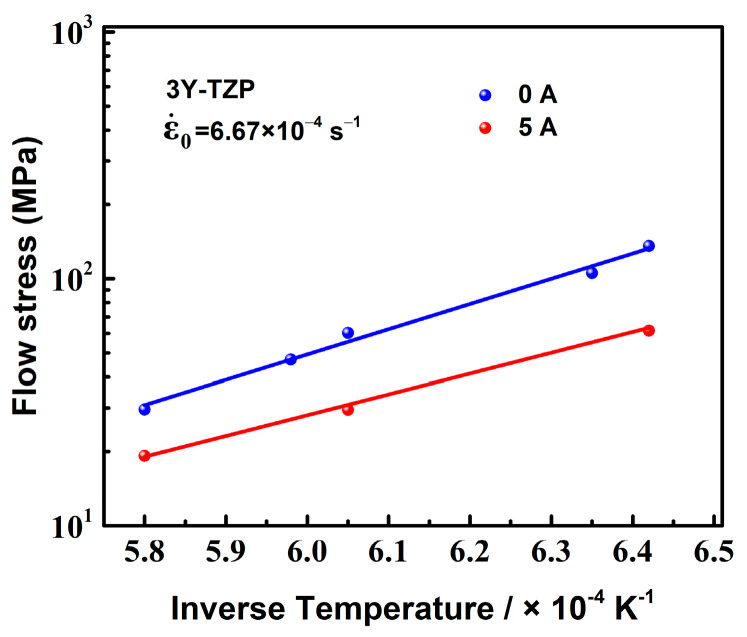
The relation between the flow stress and the inverse of the specimen temperature for the deformation with 0 A and 5 A at an initial strain rate of 6.67 × 10^−4^ s^−1^.

Generally, the specimen temperature, which can be increased by the electric current due to Joule heating, is higher than the furnace temperature [31]. In the present study, the specimen temperatures of the deformation with 5 A at the furnace temperatures of 1200 °C, 1300 °C, and 1400 °C were about 1296 °C, 1374 °C, and 1451 °C, respectively. Figure 3 shows the relation between the flow stress and the inverse of the specimen temperature for the specimens with 0 A and 5 A. The initial strain rate was 6.67 × 10^−4^ s^−1^. It is clear that the flow stress of the specimen with 5 A was significantly lower than that without the electric current, even at the same temperature. This result indicates that the application of the electric current itself also had an effect on decreasing the flow stress, in addition to Joule heating. Furthermore, for 3Y-TZP, an increased temperature of the specimen can also decrease the flow stress during conventional superplastic deformation. With an initial strain rate of 6.67 × 10^−4^ s^−1^ and the electric current of 5 A, the relative contribution of Joule heating to the decrease in flow stress at the furnace temperatures of 1300 °C and 1400 °C, which were calculated by the data in Figure 2 and Figure 3, were about 59.2% and 62.6%, respectively. Therefore, the decreased flow stress in the present study was mainly attributed to the combination of Joule heating and the applied current.

**Figure 4:**
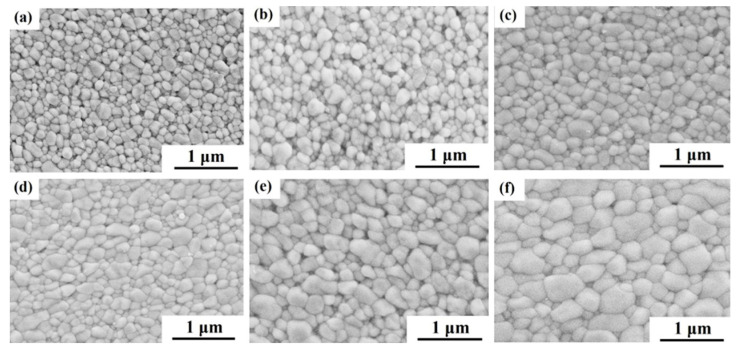
Microstructures of the deformed 3Y-TZP with/without the electric current at different furnace temperatures: (**a**) 0 A, 1200 °C; (**b**) 0 A, 1300 °C; (**c**) 0 A, 1400 °C; (**d**) 5 A, 1200 °C; (**e**) 5 A, 1300 °C; (**f**) 5 A, 1400 °C.

Figure 4 shows the cross-sectional micrographs of the deformed 3Y-TZP specimen with 0 A and 5 A at a strain rate of 6.67 × 10^−4^ s^−1^. The SEM observation points in the deformed specimen had a normalized distance from the cathode electrode of 0.3, in which the cathode and anode electrodes were located at *x* = 0 and *x* = 1, respectively. The images (Figure 1 and Figure 4) show that before and after deformation, the specimens had equiaxed grians without an obvious preferential grain growth. It can be seen that all the deformed specimens with the electric current had an accelerated grain growth, compared to those without the electric current. For example, the average grain size of the deformed 3Y-TZP specimen with 5 A was *d* = 359 nm under a strain rate of 6.67 × 10^−4^ s^−1^ at 1400 °C, while the average grain size without the electric current was *d* = 218 nm. And the average grain size of the deformed specimen in Figure 4e, for which the temperature of the specimen was estimated to be 1374 °C by the black-body radiation model [31], was larger than that without the electric current at the furnace temperature of 1400 °C. Obviously, the grain growth during the deformation was promoted by the applied electric current in the oxygen-lean atmosphere. A similar grain growth was observed in the deformation of 3Y-TZP under a flashing condition, which was reported by Yoshida et al. [19]. Tuan et al. [32] believed that the accelerated grain growth in a Y_2_O_3_-stabilized ZrO_2_ flashing condition was attributed to a reduced atmosphere.

**Figure 5:**
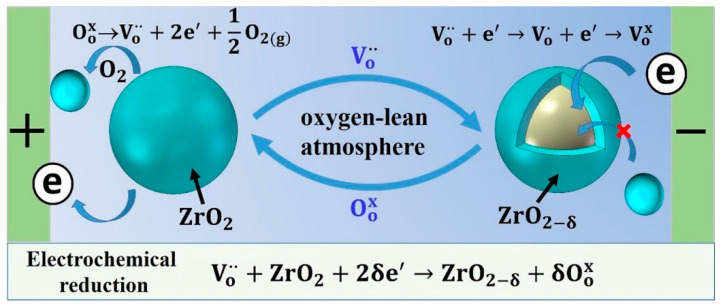
A schematic of the electrochemical reduction reaction in 3Y-TZP subjected to the current-assisted deformation. Drawn using [33] as a reference.

A previous study [13] revealed that an electrochemical reduction occurred during the electric current-assisted deformation of 3Y-TZP as a result of the oxygen-lean atmosphere, which was expressed as follows.

(5) $${\delta V}_{o}^{˙˙}+{ZrO}_{2}+{2\delta e}^{\prime }\to {ZrO}_{2-\delta }+{\delta O}_{o}^{x}$$

where $V_{o}^{˙˙}$ is the oxygen vacancies, $O_{o}^{x}$ is the oxygen ions, $O_{2(g)}$ is the molecular oxygen, and $e^{\prime }$ is the electron. In this reaction, the oxygen ions were removed from the 3Y-TZP lattice to form oxygen molecules. Correspondingly, the oxygen vacancies were incorporated into the 3Y-TZP lattice. In order to maintain the charge balance, these oxygen vacancies would capture the electrons from the cathode electrode, which caused the specimen to have a conversion from ${ZrO}_{2}$ into ${ZrO}_{2-\delta }$ (the reduced state of 3Y-TZP) [22,32,33]. A sketch summarizing the main electrochemical reduction reaction is shown in Figure 5. As the reaction proceeded (from the cathode side to the anode side), a large amount of oxygen vacancy was generated in the specimen, which accelerated the enhanced cation diffusion in the reduced Y_2_O_3_-stabilized *ZrO*_2_. And grain growth may be promoted by this enhanced cation diffusion [34,35]. Furthermore, the average grain size of the deformed 3Y-TZP with the electric current near the cathode side (*x* = 0.3, *d* = 359 nm, Figure 4f) at 1400 °C was larger than that near the anode side (*x* = 0.7, *d* = 241 nm, Figure S2). This phenomenon was consistent with that in a previous study [30]. Thus, the grain growth during deformation in the present study may be accelerated by the enhanced cation diffusion as a result of the electrochemical reduction reaction, in addition to Joule heating. And this reaction will inevitably affect the deformation mechanism.

**Figure 6:**
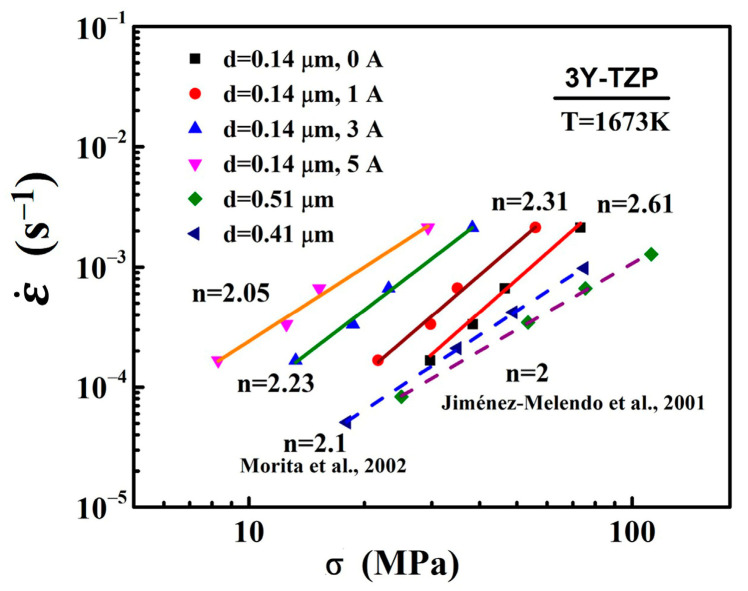
Variation of $\dot{\varepsilon }$ with σ on a logarithmic scale for specimens with the initial grain size of *d* = 0.14 μm and deformed under the electric currents of 0 A, 1 A, 3 A, and 5 A at a temperature of 1400 °C, showing a change in *n* from 2.61 to 2.05. Previously reported data [26,28] for 3Y-TZP with an d of 1.2 m are plotted for comparison.

The deformation mechanism is usually characterized by the stress exponent n and its microstructure. And the stress exponents (n) are obtained by the deformation experiments with the as-sintered grain size at the various initial strain rates. The flow stress as a function of the strain rate under different electric currents in log–log plots are shown in Figure 6. In order to decrease the influence of the grain size, the flow stress was defined as the stress at a true strain of 0.2, whereby the grain growth was related to strain [13,36]. The stress exponent n can be calculated by the plots. The stress exponent of the specimens with the different electric currents of 0 A, 1 A, 3 A, and 5 A at 1400 °C were 2.61, 2.31, 2.23, and 2.05, respectively. The stress exponent n gradually changed from 2.61 to 2.05 with an increase of the electric current. According to the literature [7,13,25,26,27,28,37], the value of the stress exponent *n* = 2 indicates that the high-temperature deformation of ceramic material was usually controlled by grain boundary sliding and the microstructure of the deformation sample was equiaxed grains. In the present study, the flow stress–strain rate relationship of *n* = 2~3 was consistent with the reported plastic flow relationship of fine-grained 3Y-TZP materials at high temperatures [7,26,27,37]. They believed that the dominant deformation mechanism was still grain boundary sliding as a result of the equiaxed grains. The SEM observations (Figure 1 and Figure 4) of the specimens revealed that before and after deformation, the samples had equiaxed grains without an obvious preferential grain growth, which is consistent with previous studies [7,26,27,37], suggesting that the dominant deformation mechanism of the specimens in the present study was grain boundary sliding. In addition, the stress exponent n of the deformation without the electric current was different from that reported in the literature [26,28], which was probably related to the grain size of 3Y-TZP before deformation. Some reports [25] have demonstrated that the stress exponent n would decrease with the grain size of 3Y-TZP before the deformation increased. Therefore, the dominant mechanism of deformation with/without the electric current in the present study was grain boundary sliding.

It is known that stress concentration will be inevitably created at the boundary ledges or multiple grain junctions during grain boundary sliding, which will cause a significant increase in the flow stress with the strain during the deformation of 3Y-TZP (strain hardening phenomenon) [28]. As shown in Figure 4b, an apparent strain hardening occurred during the deformation without the electric current. With the electric current applied, the flow stress during deformation decreases and the apparent strain hardening was significantly eliminated. And the variation of the stress exponent *n* (from 2.61 to 2.05) suggests that the limitation of grain boundary sliding during the deformation was decreased. Recently, Charalambous et al. [38] found that the grain boundary was weakened by the point defects (oxygen vacancy and reduced phases) generated by the electrochemical reduction reaction in the flash-sintered Y_2_O_3_-stabilized ZrO_2_. Some recent reports [39,40] have pointed out that the grain-boundary sliding rate during deformation could be promoted by these weakened grain boundaries as a result of the generated point defects. Therefore, the electric current applied to 3Y-TZP may promote grain boundary sliding by the same mechanism.

**Figure 7:**
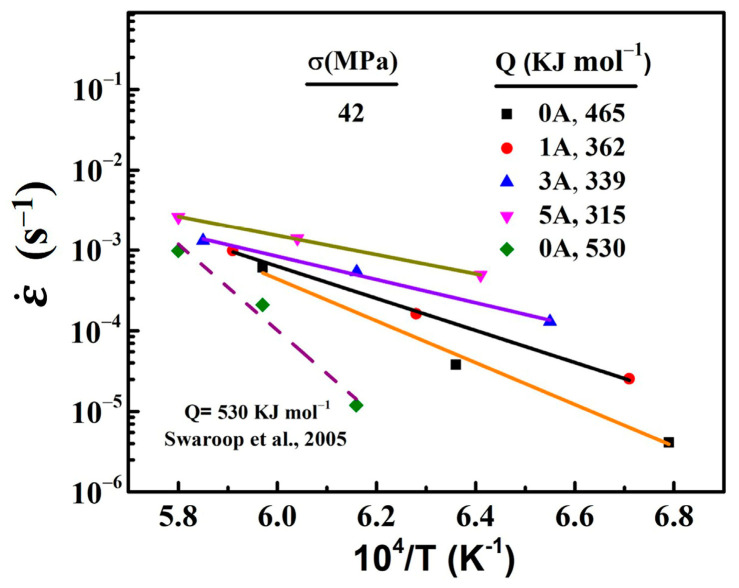
The effect of the temperature on the strain rate at 42 MPa. Previously reported data [45] for YTZP are plotted for comparison.

The relationship between the temperatures and strain rates are shown in Figure 7. The apparent activation energy *Q* was calculated from the slope lines. The apparent activation energies of 0 A, 1 A, 3 A, and 5 A were 465, 362, 339, and 315 kJ mol^−1^, respectively. It can be seen clearly that there was a significant decrease in the activation energy as the electric current was applied. And the apparent activation energy decreased as the electric current increased. Generally, the apparent activation energy is interpreted as the energy for the Zr cation diffusion of the high-temperature plastic flow in 3Y-TZP [41]. In this study, the decrease in the activation energy by the electric current may be due to a decrease in the energy for the formation and/or migration of Zr cation vacancy. Several research groups [18,33,38,42] have reported that, under flash-sintering conditions or in an oxygen-lean atmosphere, large amounts of oxygen vacancies were generated by the electrochemical reduction. The first-principles calculation for Y_2_O_3_-stabilized ZrO_2_, worked on by Chen et al. [43], indicates that the formation of the oxygen anion vacancy favored a decrease of the migration barrier of the Zr cation vacancy and enhanced the diffusion of Zr cations. In addition, A molecular dynamics study of Y_2_O_3_-stabilized ZrO_2_ showed that an electric field of 500~1000 V·cm^−1^ could induce the formation of cation vacancy and increase the cation vacancy concentrate at the grain boundaries, which enhanced the diffusivities of cations and anions [44]. Therefore, the decrease in the apparent activation energy *Q* during the deformation was attributed to the point defects generated by the electric current.

In addition, cation diffusion is usually regarded as the rate-controlling step for the superplastic deformation of Y-TZP [41]. Some studies [5,45] have reported that the activation energies for the grain boundary diffusion and lattice diffusion of Zr cation in 3Y-TZP are around 370 kJ mol^−1^ and 500 kJ mol^−1^, respectively. The activation energy for the deformation of 3Y-TZP without an electric current (*Q* = 465 kJ mol^−1^), which was close to the activation energy for the lattice diffusion of Zr cation, indicated that the deformation rate of 3Y-TZP was controlled by the lattice diffusion of Zr cation. The decrease in the apparent activation energy by the applied electric current indicated that the lattice diffusion of Zr cation was enhanced. And the deformation rate of 3Y-TZP with the electric current may be controlled by the grain boundary diffusion of Zr cation.

## 4. Conclusions

The compression deformation of 3Y-TZP ceramics with different electric currents in an oxygen-lean atmosphere was investigated. The results are summarized as follows:

1. The superplastic deformation of 3Y-TZP was improved by the electric current in an oxygen-lean atmosphere. With the electric current increased, the flow stress during deformation had a significant decrease. And this decreased flow stress resulted from a combined effect of Joule heating and the enhancement of the Zr cation diffusion due to the electric current. The relative contribution of Joule heating indicated that Joule heat had a more significant effect on decreasing the flow stress in a deformation with 5 A.
2. Significant grain growth was observed in the deformed specimen with the electric current. And the microstructures of the deformed specimens were all equiaxed grains without an obvious preferential grain growth. The stress exponents n of the deformation with different electric currents at 1400 °C were determined to be *n* = 2.05~2.61, which suggested that the dominant deformation was grain boundary sliding.
3. The apparent activation energies for the deformation with different electric currents were evaluated to be about 315~365 kJ·mol^−1^, which was significantly lower than without the electric current (465 kJ·mol^−1^). This decreased apparent activation energy indicated that the cation diffusion was accelerated during the deformation by the electric current, which may result in a transition of the rate-controlling step for the deformation from lattice diffusion to grain boundary diffusion.

## Data Availability

Not applicable.

## Supplementary Materials

The following supporting information can be downloaded at: https://www.mdpi.com/article/10.3390/ma16206785/s1, Figure S1: A schematic diagram of the apparatus used for the experiment. Figure S2: The microstructure of the deformed 3Y-TZP with 5 A near the anode side (x = 0.7) at 1400 °C, in which the cathode and anode electrodes were located at x = 0 and x = 1, respectively. Table S1: The grain sizes of 3Y-TZP before and after deformation at a true strain of 0.5 and at different deformation temperatures.
