# Supplementary material for: Effects of an Electric Current on the Superplastic Deformation Behavior of 3Y-TZP in an Oxygen-Lean Atmosphere
Source: Materials (Basel). 2023 Oct 20;16(20):6785. doi: 10.3390/ma16206785 (PMC10607986; doi:10.3390/ma16206785)
Supplement: Supplementary file 1 [file materials-16-06785-s001.zip › materials-2673926-supplementary.pdf]

Effects of an Electric Current on the Superplastic Deformation Behavior of 3Y-TZP in an Oxygen-Lean Atmosphere

Kang Wang [1], Yufei Zu [2], Guoqing Chen [1,*], Xuesong Fu [1] and Wenlong Zhou [1]

[1] Key Laboratory of Solidification Control and Digital Preparation Technology (Liaoning Province), School of Materials Science and Technology, Dalian University of Technology, Dalian 116085, China; wangkang245@mail.dlut.edu.cn (K.W.); xsfu@dlut.edu.cn (X.F.); wlzhou@dlut.edu.cn (W.Z.)
[2] Key Laboratory of Advanced Technology for Aerospace Vehicles (Liaoning Province), School of Aeronautics and Astronautics, Dalian University of Technology, Dalian 116085, China; yfzu@dlut.edu.cn
* Correspondence: gqchen@dlut.edu.cn; Tel.: +86-0411-84707970

Supplementary material

A schematic diagram of the apparatus used for the experiment is shown in Figure S1.

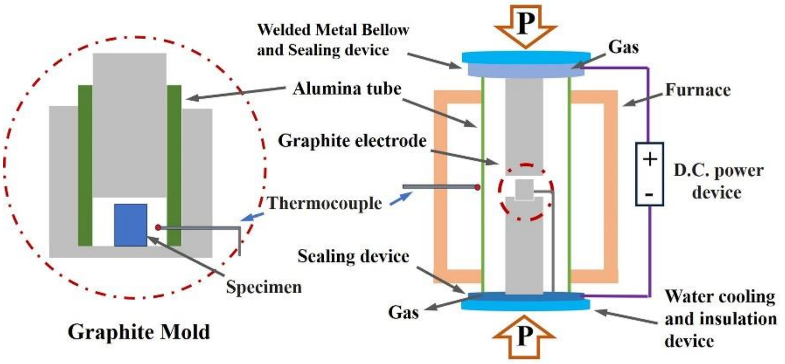

Figure S1. A schematic diagram of the apparatus used for the experiment.

Figure S2 shows the microstructure of the deformed 3Y-TZP with 5 A near the anode side（x = 0.7）at 1400℃, in which the cathode and anode electrodes were located at x = 0 and x = 1, respectively. The corresponding average grain size was 241 nm.

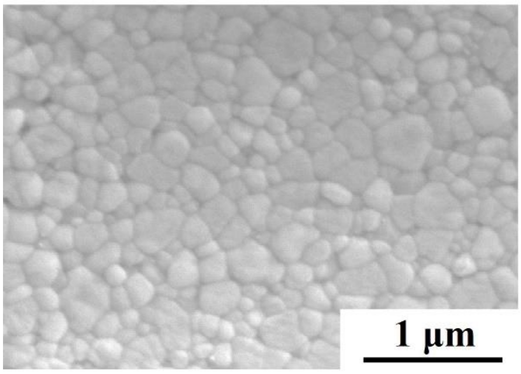

Figure S2. The microstructure of the deformed 3Y-TZP with 5 A near the anode side （x = 0.7） at 1400℃, in which the cathode and anode electrodes were located at x = 0 and x = 1, respectively.

The grain sizes of 3Y-TZP before and after deformation at a true strain of 0.5 and at different deformation temperatures are shown in Table S1.

Table S1. The grain sizes of 3Y-TZP before and after deformation at a true strain of 0.5 and at different deformation temperatures.

Temperature (℃)	Initial grain size (nm)	Grain size before deformation (nm)	Grain size after deformation with 0 A (nm)	Grain size after deformation with 5 A (nm)
1200 ℃	125	125	159	209
1300 ℃	125	136	191	265
1400 ℃	125	148	218	359

The SEM observation points in the deformed specimen had a normalized distance from the cathode electrode of 0.3, in which the cathode and anode electrodes were located at x = 0 and x = 1, respectively.
